# Women's attitude towards prenatal screening for red blood cell antibodies, other than RhD

**DOI:** 10.1186/1471-2393-8-49

**Published:** 2008-11-11

**Authors:** JM Koelewijn, TGM Vrijkotte, M de Haas, CE van der Schoot, GJ Bonsel

**Affiliations:** 1 Sanquin Research, Amsterdam, and Landsteiner Laboratory, Academic Medical Centre, University of Amsterdam, Plesmanlaan 125, 1066 CX Amsterdam, The Netherlands; 2 Academic Medical Centre, Department of Social Medicine, Meibergdreef 9, 1105 AZ Amsterdam, The Netherlands; 3 Institute Health Policy and Management, Erasmus Medical Centre, Postbox 1738, 3000 DR Rotterdam, The Netherlands

## Abstract

**Background:**

Since July 1998 all Dutch women (± 200,000/y) are screened for red cell antibodies, other than anti-RhesusD (RhD) in the first trimester of pregnancy, to facilitate timely treatment of pregnancies at risk for hemolytic disease of the fetus and newborn (HDFN). Evidence for benefits, consequences and costs of screening for non-RhD antibodies is still under discussion. The screening program was evaluated in a nation-wide study. As a part of this evaluation study we investigated, according to the sixth criterium of Wilson and Jüngner, the acceptance by pregnant women of the screening program for non-RhD antibodies.

**Methods:**

Controlled longitudinal survey, including a prenatal and a postnatal measurement by structured questionnaires. Main outcome measures: information satisfaction, anxiety during the screening process (a.o. STAI state inventory and specific questionnaire modules), overall attitude on the screening program. Univariate analysis was followed by standard multivariate analysis to identify significant predictors of the outcome measures. Participants: 233 pregnant women, distributed over five groups, according to the screening result.

**Results:**

Satisfaction about the provided information was moderate in all groups. All screen- positive groups desired more supportive information. Anxiety increased in screen- positives during the screening process, but decreased to basic levels postnatally. All groups showed a strongly positive balance between perceived utility and burden of the screening program, independent on test results or background characteristics.

**Conclusion:**

Women highly accept the non-RhD antibody screening program. However, satisfaction about provided information is moderate. Oral and written information should be provided by obstetric care workers themselves, especially to screen-positive women.

## Introduction

The scope of prenatal screening has considerably widened last two decades. The number of tests increased, and the time frame expanded to preconceptional. While consensus exists about the restriction to evidence-based tests for routine use, the benefits and burden of many tests in current use are poorly documented, as is the case for screening for red blood cell (RBC) antibodies, other than Rhesus-D (RhD).

Screening for non-RhD antibodies in all pregnant women has been implemented in most developed countries. In the Netherlands, screening for those so called non-RhD antibodies, was introduced in 1998 in absence of evidence of its effectiveness and costs [1,2].

Clinically relevant non-RhD antibodies can cross the placenta and may, like RhD antibodies, induce hemolytic disease of the fetus and newborn (HDFN). HDFN is a serious condition that can give rise to fetal hydrops, fetal death or neonatal hyperbilirubinemia, resulting in permanent neurological damage by kernicterus.

The obvious objective of the non-RhD screening program is timely detection of pregnancies at risk of severe HDFN, as this condition can be effectively treated by intra uterine transfusions and/or postnatal exchange transfusions in severe cases, or by postnatal phototherapy and/or blood transfusions in moderate cases [3-5]. Moreover, screening during pregnancy facilitates quick identification of the specificity of detected antibodies, if a blood transfusion to the mother is necessary during delivery.

Despite the face validity of this approach, which facilitated its introduction, empirical evidence is limited compared to the evidence supporting screening for RhD antibodies. For this reason the Dutch screening program was evaluated in a nation-wide study [6]. The results of this study show that, if we compare screening for non-RhD antibodies and for RhD antibodies, the prevalence of non-RhD antibodies is about fourfold (328/100,000 versus 75/100,000). However, the number needed to screen (NNS) do detect severe HDFN, due to non-RhD antibodies is 20,000, compared to 4,000 to detect severe HDFN by RhD antibodies This is due to two reasons. First, many pregnant women show non-RhD antibodies due to previous blood transfusions (transfusions are RhD matched). For this reason in about 40% of the non-RhD positive pregnancies the father – and also the fetus – is antigen-negative for the blood group antigen against which the maternal antibodies are directed; in these cases the fetus is not at risk of developing HDFN [6]. In case of RhD antibodies almost all fathers are antigen-positive, which underlies the observed immunization [7]. Second, among many non-RhD antibodies, only few (only anti-K, anti-c, anti-C, anti-e and anti-E) actually can cause severe HDFN [3,6]. Combining probabilities it turns out that about 1:50 of pregnancies with non-RhD antibodies results in severe HDFN versus 1:4 of pregnancies with RhD-antibodies [6].

Because of the high NNSs of the non-RhD screening program compared to RhD screening, the acceptance of the non-RhD screening program by pregnant women, a prerequisite following the Wilson & Jüngner criteria [8], is in particular important.

This paper explores the attitude towards the screening program among several groups of pregnant women, relating acceptance to being informed and experienced burden. Also it reports the experienced burden of the screening process in all its stages. This report is part of a nation-wide evaluation study on non RhD screening to address expressed public and professional concern on this prenatal program.

## Methods

### National screening program

RBC antibody screening is part of the booking visit protocol, and free of charge. The obstetric care worker (independent midwife 75%, general practitioner 5%, obstetrician 20%, [9] is responsible. The screening test is performed by local laboratories (n = ± 90), resulting in 1.2% positive screening results. Blood of screen-positives is sent to one of two specialized national reference laboratories. After confirmation of the positive screen result (20% is not confirmed), the reference laboratory determines the antibody specificity. In 47% of screen-positive pregnancies clinically non-relevant antibodies are found (not able to cross the placenta, so not causing HDFN), in 33% clinically relevant (HDFN causing) antibodies are detected. [6] In those pregnancies the paternal antigen pattern is typed, because only if the father is antigen-positive, the fetus is at risk of HDFN. Pregnancies at risk are monitored by repeated laboratory testing in the reference laboratories. If testing suggests a major risk of HDFN, women are clinically monitored, by ultrasound, Doppler flow measurement and, rarely, by amniocentesis (fetal antigen genotyping and/or Liley Index). The most severe cases of HDFN are monitored and – if necessary – treated with intra uterine transfusions in one expert centre [10].

### Study design

A controlled longitudinal survey was performed, including a prenatal measurement around week 20 and a postnatal measurement two weeks after birth, using structured questionnaires.

To create a longitudinal perspective on both occasions questions referred to actual experiences and experiences in the preceeding relevant period.

**Figure 1:**
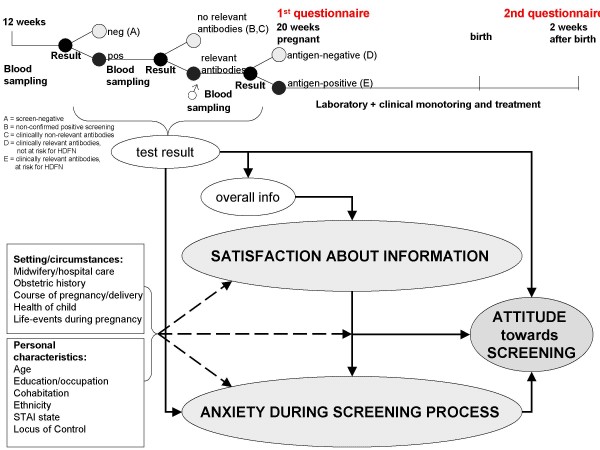
Behavioral/attitudinal model screening for non RhD RBC antibodies.

The set of questions was based on a prespecified behavioral model (Figure 1).

The study proposal was judged by the Ethical Committee of the Academic Medical Centre in Amsterdam. This Committee established that this study design did not require formal approval of the Committee.

All participants gave informed consent; at least moderate command of the Dutch language was required.

### Participants

Women from the following groups, ranked according to the result of the screening and the confirmation test, were included:

- Group A) controls (screen-negative);

- Group B) women with a positive screening result, however not confirmed by the reference laboratory; only women, aware of their screen-positivity, were included; during the study it appeared that many care workers did not reveal the initial screen-positive test to the woman;

- Group C) women with clinically non-relevant RBC antibodies (i.e. antibodies that cannot cause HDFN);

- Group D) women with clinically relevant non RhD antibodies, nevertheless not at risk of HDN because the father was antigen-negative;

- Group E) women with clinically relevant non-RhD antibodies, at risk of HDFN (father antigen-positive).

Women in group D and E also participated in the nation-wide evaluation study of the non-RhD screening program.

We used a quota sampling method and intended at onset to select at least 50 controls under midwifery care in the first trimester of pregnancy and 50 controls under care of an obstetrician (hereafter referred to as 'hospital care'/'hospital practices'), as well as 50 women from each of the screen-positive groups (B, C, D, E). We expected that 50% of the selected women would complete both questionnaires.

Pregnant women were enrolled by the obstetric care worker during the second prenatal visit or, if screen-positive, as soon as the results of the confirmation test in the reference laboratories, as well as the paternal antigen typing, were known. Excluded were women with a miscarriage before the moment of inclusion.

Controls were selected in 23 randomly selected midwifery practices (response 23/28 = 82%) and 17 hospital practices (response 17/22 = 77%) in May-June 2005.

Midwives were asked to select four and obstetricians to select two or – if possible – three random controls on the base of having a similar date of communication of the screening test result to the pregnant woman (or coming closest to that date). Screen-positive women (groups B, C, D, E) were consecutively identified at the two reference laboratories from April-June 2005 and included via the obstetric care worker. The numbers of eligible women who were actually asked by the obstetric care worker to participate in the study by the obstetric care worker, were not registered in the groups of women without clinically relevant antibodies (A, B and C).

Command of the Dutch language was at the judgement of the obstetric care worker.

### Behavorial/attitudinal model

A conceptual model was developed ex ante to elucidate the relation between the process of antibody screening, personal maternal characteristics, setting of care and maternal circumstances, satisfaction about the provided information, anxiety during the screening process, and the overall attitude towards the screening. (Figure 1). The model was based on similar models that are used in the evaluation of (pregnancy) screening [11-14].

Two validated questionnaires were used:

- Multidimensional Health Locus of Control (MHLC), consisting of 18 items, distributed over three subscales with each six items: 'internal', 'chance' and 'powerful others'. MHLC sum scores were calculated per subscale. If more than two items were missing within one subscale, the subscale score was missing. One or two missing items were substituted by the mean of the non-missing items in the subscale [15].

- Spielberger's State-Trait Anxiety Inventory Questionnaire (STAI), to measure general background anxiety (STAI trait) and general situational anxiety (STAI state). Each questionnaire consists of 20 items with four response categories with a range of 20–80. The STAI-scores were only calculated if at least 17 items (out of 20) were filled out. For three or less missing items a value of 2.0 was substituted [16,17].

Specific questionnaires were developed for this project, based on existing formats:

- Knowledge on RBC antibodies and HDFN was derived from the response to 17 yes/no questions.

- Screen-related anxiety at different stages of the screening process was captured by eliciting self report data on the level of anxiety before the test result (screening, reference laboratory confirmation test, paternal antigen typing, laboratory monitoring) was known, and on the level of relief, reassurance and fright after hearing the test result. Women had to tick a cross on a visual analogue scale (VAS) of 160 mm with four anchors (not at all (0 mm) – a little bit (55 mm) – rather (110 mm) – very much (160 mm)).

- Satisfaction about the availability and comprehensibility of information, and the overall judgment of the screening process were measured using a similar four anchor VAS (very unsatisfied (0 mm) – unsatisfied (55 mm) – satisfied (110 mm) – very satisfied (160 mm)).

VAS are widely used in health care research, for example for scaling of ranked differences between health states, and for scaling of anxiety [18,19].

(all materials available from the first author).

### Data collection

The prenatal questionnaire covered the MHLC, STAI trait and STAI state, knowledge on RBC antibodies, screen-related anxiety and satisfaction about provided information until this moment. This questionnaire additionally recorded personal characteristics and data on the obstetric history. The postnatal questionnaire measured the course of pregnancy and delivery, the STAI state, screen-related anxiety, satisfaction about information, and the overall judgement about the screening program.

All questionnaires were tested in a pilot study among cases with clinically relevant antibodies and controls (data not used in this study).

The prenatal questionnaire was offered by the obstetric care worker around week 20, about 8 weeks after screening, and completed within a week, the postnatal questionnaire was sent by post to the mother twelve days after the actual date of birth or – if the date of birth was unknown – after the expected date of birth. The women were instructed to complete the questionnaire 14 days after the actual day of birth. If this was not possible, the woman was instructed to recall to mind her situation 14 days after birth.

### Data analysis

Prior to analysis the data of the controls under hospital care were linearly weighted (factor 0.41)) to accommodate the actual 80%-20% balance of primary care worker and secondary care worker involvement in early pregnancy. So doing, we excluded selection bias effects, as in the Dutch obstetric organisation obstetric risk factors are more prevalent in women under hospital care, and obstetric risk factors as well as the setting of care may be related to other background characteristics (for example anxiety), influencing our main outcome measures.

Our behavorial/attitudinal model focuses on (1) information satisfaction, (2) anxiety, and (3) overall attitude towards the screening program, comparing the responses of the predefined groups. For all three outcomes the relevant cross table or figure is followed by standard exploratory multivariate analysis. Variables with a p-value <0.10 in the univariate analysis were offered forward to the multivariate model according to the time sequence of the occurrence of these risk factors; models were checked for interaction.

The VAS scores were measured in millimetres (from 0–160) and divided through 16 to achieve a score from 0 until 10. A score of 0 reflects 'not at all', 3.4 ' a little bit', 6.9 'rather' and 10 'very much'.

Specific screen-related stress was distinguished in anxiety before a test result was known and in the impact of the result of a specific test. Anxiety was calculated as the mean VAS score on 'anxiety about the test result' and 'thinking about the test result'. The impact of the test result was calculated from three items concerning the feelings of the mother after hearing the test result: relief, reassurance and fright according to the following formula: fright – MEAN (relief, reassurance).

Satisfaction about information provided by the obstetric care worker, was summarized in one sum score, calculated as the mean of:

- the mean score on all questions regarding the amount of information provided by the obstetric care worker, on different moments during the screening process

- the mean score on all questions regarding the comprehensibility of information, provided by the obstetric care worker, (verbal and written) on different moments during the screening process

- if there is a match between the mode of reporting the screening result: 7; if there is no match: 3.

In the relevant cross tables outcomes, scored on a VAS, are presented in three categories (VAS score < 3.4, VAS score ≥3.4 < 6.9, VAS score > 6.9), and analysed as categorical variable as well as continuous variable. In the multivariate analysis the continuous VAS scores are used.

All data were analysed with SPSS 11.0. Differences between categorical variables were tested by Pearson's chi square test or Fisher's exact test. Differences between means of continuous variables were tested by ANOVA and by the Student's t-test. A difference was considered as significant if the p-value was < 0.05. The value in each group was tested against the controls.

## Results

### Response

The response in women with clinically relevant antibodies (D and E) was 90%. In the groups without clinically relevant antibodies (A, B and C) the response rate was unknown. In each group at least 50 women were selected. The prenatal questionnaire was completed by 263; 233 (89%) also completed the second questionnaire (Table 1). In group B (positive screening, unconfirmed) less than 25 women completed both questionnaires. The reasons for not completing the second questionnaire were unknown.

**Table 1:** Response according to HDFN risk, as established upon first trimester RBC antibody screening

**Groups according to risk for HDFN**	**Questionnaire 1 completed** **no**	**Questionnaire 1 + 2 completed** **no (%)**
A1. controls midwifery practice, screening negative	61	58 (95)
A2. controls hospital practice, screening negative	41	35 (85)
B. positive screening result, not confirmed	24	21 (88)
C. clinically non-relevant antibodies	48	44 (92)
D. clinically relevant non-RhD antibodies, not at risk HDFN (father antigen-negative)	37	30 (81)
E. clinically relevant non-RhD antibodies, at risk HDFN (father antigen-positive)	52	45 (87)
Total	263	233 (89)

No significant differences in response between the groups

### Baseline characteristics

Table 2 outlines the baseline characteristics of the respondents, according to the risk of HDFN, as established upon the first trimester antibody screening. Cases at risk (group E) differed from the other groups related to known risk factors for antibody development [4]: a.o. cases were more often multiparous, were older and were more often under hospital care in early pregnancy. Cases at risk (group E) reported more often women with RBC antibodies in their environment, and had more knowledge about RBC antibodies.

**Table 2:** Baseline characteristics, respondents grouped according to HDFN risk, as established upon first trimester RBC antibody screening

	Controls	Screen-positive, not at risk HDFN			At risk HDFN
	n = 73^3^	Screen-pos, not-confirmed n = 21	Clinically non-relevant antibodies n = 44	Non-RhD antibodies, father neg. n = 30	Non-RhD antibodies, father pos. n = 45

***General***					
Age – yr mean (SD)	30.6 (4.2)	31.3 (3.7)	30.5 (4.8)	30.6 (4.2)	32.9 (3.4)*
Educational level – %					
. elementary or lower secondary school	14	9	23	40*	9
. higher secondary school	37	43	36	30	49
. higher vocational level/university	49	48	41	30*	42
Ethnic group: non-Dutch – %	4	0	14	0	0
MHLC^1 ^(range 6–36) – mean (SD)					
Domains:					
- Internal	22.1 (4.1)	22.5 (3.4)	21.7 (3.7)	21.5 (5.2)	21.7 (3.6)
- Chance	17.6 (3.6)	17.2 (4.8)	16.7 (3.9)	17.9 (5.6)	18.2 (4.5)
- Powerful others(doctors)	13.9 (3.6)	13.8 (3.4)	14.7 (4.0)	13.8 (4.0)	15.2 (4.1)
STAI trait – mean (SD)	34.0 (7.3)	31.4(6.3)	34.8 (8.3)	34.1 (7.8)	33.7 (7.9)

***Disease specific experience***					

Known with RBC antibodies/HDFN – %	NA	NA	23	30	27
RBC antibodies in environment – %	26	29	30	17	44*
HDFN in environment – %	10	10	0*	7	16
Knowledge about RBC antibodies (range:0–17) – mean (SD)	7.6 (4.1)	8.4 (3.5)	8.4 (4.0)	9.2 (4.1)	11.7 (3.4)*

***Obstetric experience***					

History of former pregnancy/delivery – %	51	62	66	63	96*
. former pregnancy/delivery with problems – %	19	10	23	10	33
Care during current pregnancy – %					
- start in primary care	80	86	77	63	42*
- primary → sec care (% of) started in primary care)	35	39	29	32	42
Hospital admission in current pregnancy – %	14	10	25	23	22
Non-medical life-events in current pregnancy – %	43	33	43	37	27
Invasive procedure current delivery^2 ^– %	33	29	21	30	27
Bad experience current delivery – %	18	33	21	23	11
Not well (mother and/or child) 14 days after current delivery – %	12	14	7	21	4

^1 ^Multidimensional Health Locus of Control Scales

^2^Invasive procedure delivery: assisted vaginal delivery, cesarean section, manual removal of placenta, surgical suturing

^3^Weighted controls from obstetric care: 35*0.41 = 15; 58 controls from midwifery care

* p < 0.05, compared to controls

### Availability of information; satisfaction with information

Table 3 outlines the availability and use of information. Only half of the women in the groups A, B, C and E had actually read the written information about RBC antibody screening, which generally is provided by care workers. In group D significantly more women (77%) reported to have read the written information. The role of oral professional information increased with risk status: all women with at risk of HDFN (group E) were informed orally. The use of internet information showed the same pattern.

**Table 3:** Received information about antibodies and HDFN, according to HDFN risk, as established upon first trimester RBC antibody screening

	Controls	Screen-positive, not at risk HDFN			At risk HDFN
	n = 73^1^	Screen-pos, not-confirmed n = 21	Clinically non-relevant antibodies n = 44	Non-RhD antibodies, father neg. n = 30	Non-RhD-antibodies, father positive n = 45
*Mode of reporting result screening for RBC antibodies*					
- regular consult – %	84	67	73	64*	36*
- telephone	4	29*	16*	23*	44*
- letter	3	4	9	10	9
- other way	1	0	2	3	7
- not reported	8	0	0	0	4
*Information read in written information, provided by obstetric care worker?*					
Yes – %	51	43	55	77*	49
*Verbal information provided by obstetric care worker*					
Yes – %	65	76	84*	90*	96*
*Information sources found by the woman herself* (more than one source possible)					
- written – %	45	19*	21*	33	16*
- internet	10	19	27*	30^≈^	53*
- friends/family	21	14	11	23	27
- education	18	24	5*	7	7
- OPZI-study ^2^	0	0	0	23*	13*
- other sources	4	5	0	0	7

^1 ^Weighted controls from hospital care: 35*0.41 = 15; 58 controls from midwifery care

^2 ^OPZI-study: nation-wide evaluation of the first screening program for RBC antibodies, other than RhD

* p < 0.05, differences compared to controls

The majority of women, regardless the group with clinically non-relevant RBC antibodies (group C), had a fairly accurate account of the interpretation of results of the subsequent antibody blood tests (detailed data available on request). Only three of 93 controls erroneously thought that RBC antibodies were present in their case. One woman out of 21 with a non-confirmed positive screening (group B) and nine women of 44 (20%) with clinically non-relevant antibodies (group C) thought that they had antibodies that for certain could harm the child.

Table 4 presents selected data on the satisfaction with the information provided by the obstetric care worker. Personal communication, either at the office or by telephone, was the strongly preferred mode of communication of the screen result, regardless the risk status. In retrospect the probability of a mismatch between preferred and actual mode of communication increased according to risk status from 16% in group A) to 38% in group E). Group E) differed from the other screen-positive groups (B, C, D) in the choice between information at the office versus by telephone, where no difference should be expected: this survey question refers to a stage where women do not know to which group they will belong. The difference can be explained by post hoc adjustment: once you know you are at risk you might prefer in retrospect to have heard this by telephone rather than at the next consult.

**Table 4:** Satisfaction about information on RBC antibodies and HDFN, according to HDFN risk, as established upon first trimester RBC antibody screening

	**Controls**	**Screen-positive, not at risk HDFN**			**At risk HDFN**
	**n = 73**^**1**^	**Screen-pos, not-confirmed** **n = 21**	**Clinically non-relevant antibodies** **n = 44**	**Non-RhD antibodies, father neg.** **n = 30**	**Non-RhD antibodies, father pos.** **n = 45**

***Info from obstetric care worker***					
- amount of info					
. dissatisfied^2 ^-%	3	5	5	13	4
. not really satisfied	82	76	86	64	71
. satisfied	15	19	9	23	25
- comprehensibility info					
. dissatisfied -%	1	5	5	3	2
. not really satisfied	82	62	90	74	67
. satisfied	17	33	5	23	31
***Preferred mode of communication result of RBC antibody screening***					
- regular consult -%	93	57*	77*	90	60*
- telephone	3	24*	9	10	40*
- letter	3	14	7	0	0
- other way	1	5	7	0	0
Mismatch of preference versus actual mode of communication -%	16	19	30	30	38*
***Sumscore satisfaction on information from obstetric care worker*[**					
. dissatisfied^2 ^-%	3	5	7	10	4
. not really satisfied	65	76	70	73	80
. satisfied	32	19	23	17	16
*Wish for more info 2 weeks after birth *-%	21	67*	54*	43*	69*
. written	18	62*	47*	37*	53*
. verbal	8	19	21*	20	27^»^
. internet	3	33*	9	10	20*
. other way	1	10	5	10	11*
-**Topics about which more information is wished (More than one topic possible)**					
. consequences child -%	15	57*	46*	33*	60*
. consequences mother	14	57*	41*	33*	47*
. treatment child	8	24	18	13	47*
. next pregnancy	7	33*	25*	20	42*
. blood testing	10	38*	16	13	24*
. other topics	0	5	0	0	11*

^1 ^Weighted controls from hospital care: 35*0.41 = 15; 58 controls from midwifery care

^2 ^Dissatisfied: VAS score < 3.4; not really satisfied: VAS score between 3.4 and 6.9; satisfied: VAS score >= 6.9 (see methods)

* p < 0.05, differences compared to controls

On average the satisfaction about the amount of and the comprehensibility of provided information was moderate in all groups. All screen-positive groups (B, C, D, E) desired more supportive (preferably written) information, prenatally (data not shown; not different from postnatal data) and postnatally. Not one singular topic in need of information prevailed.

In the multivariate analysis (Table 5) the summary score on satisfaction about information was significantly correlated with the STAI trait (the more anxious, the more dissatisfied), referral from primary to secondary care during pregnancy (after referral more satisfied), and being screen-positive (screen-positives more dissatisfied). The desire for more information (not part of the summary score) was also correlated with screen-positivity (screen-positives wanted more info) and the STAI state in week 20 (the more anxious, the more desire for information).

**Table 5:** Significant predictors (p < 0.05) of main outcome measures on RBC antibody screening in multivariate analysis

	Satisfaction on information		Anxiety 2 weeks after birth		Balance usefulness-burden 2 weeks after birth	
	sumscore info satisfaction	wish more info 2 weeks after birth	pregnancy-related anxiety	general anxiety: STAI state	for myself	for others
	Standardized β	Exponent B	Standardized β		Standardized β	

STAI trait	- 0.20		0.16	0.41		
STAI state 20 weeks		1.04				
HDFN in environment			0.14			
Screen-positivity	-.0.17	5.1				
At risk HDFN (group E)					0.143	
Referral during pregnancy	0.17					
Invasive procedure delivery			0.18			
Mother and/or child not well 2 weeks after birth			0.40	0.31		

R square	0.09	0.16	0.29	0.28	0.02	

### Anxiety

**Figure 2:**
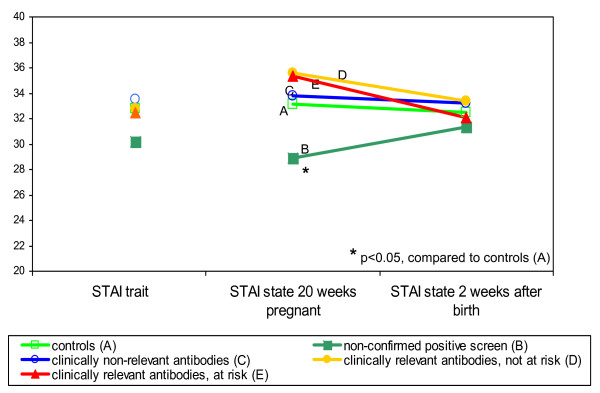
General anxiety, according to HDFN risk status.

General background anxiety, as measured by the STAI trait, was not related to the result of the screening test. The STAI state in week 20 was lower in the group (B) with an unconfirmed positive screening (28.9), compared to the other groups (scores from 33.2 until 35.6)); after birth no difference was found between the groups (Figure 2).

**Figure 3:**
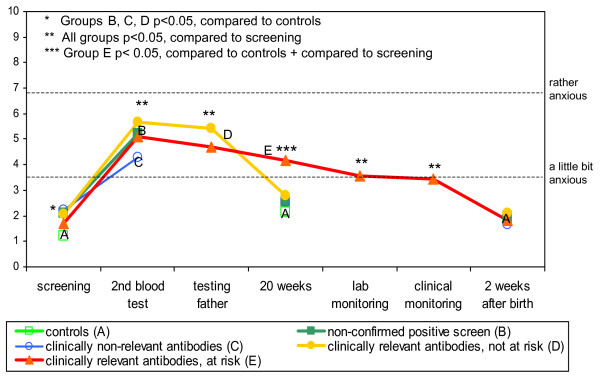
Specific screening related anxiety during screening process until 2 weeks after birth, according to HDFN risk status.

Specific anxiety (4 anchor VAS) apparently was related to the screening process (Figure 3). Scores increased after the positive screening result, waiting for the second blood test, to a level between 'a little bit anxious' and 'rather anxious'. From then onwards screen-related anxiety attenuated to baseline (< 'a little bit anxious') in all groups. Laboratory and clinical monitoring apparently reduced rather than induced anxiety.

**Figure 4:**
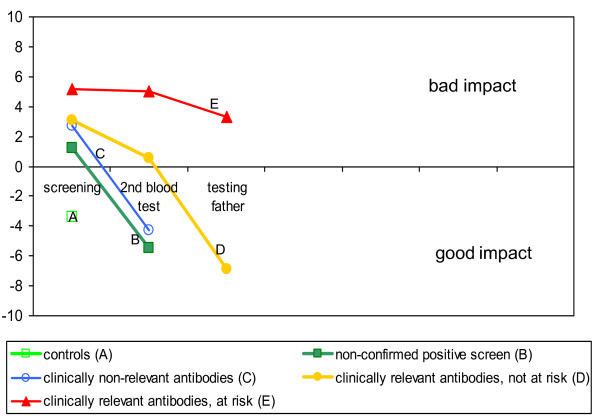
Impact of results of blood testing to determine HDFN risk status, according to risk status.

Our summary score on test result impact showed a 'bad' impact (sumscore < 0, implying that the test results causes more fright than reassurance and relief) of a positive test result, and a 'good' impact (sumscore > 0) if no risk of HDFN turned out to be present (Figure 4).

In the multivariate analysis (Table 5) anxiety about mother and child two weeks after birth was significantly correlated with the condition of mother and child at that moment (more anxiety if mother and/or child were not well), invasive procedures during delivery (cause more anxiety), STAI trait (a higher score predicted more anxiety), and the presence of HDFN in the environment (predicted more anxiety). The postnatal STAI state was correlated with essentially the same variables: STAI trait and the current condition of mother and child.

### Overall attitude

The majority of women considered the burden of the non-RhD antibody screening as low, prenatally as well as postnatally, however with significant higher scores in screen-positive women (Table 6). Most women in all groups considered the non-RhD antibody screening as useful; only women with clinically non-relevant antibodies more frequently considered the screening as not useful. See Table 6.

**Table 6:** Opinion about burden and usefulness of RBC antibodies and HDFN 2 weeks after birth, according to HDFN risk, as established upon first trimester RBC antibody screening

	**Controls**	**Screen-positive, not at risk HDFN**			**At risk HDFN**
	**n = 73**^**1**^	**Screen-pos, not-confirmed** **n = 21**	**Clinically non-relevant antibodies** **n = 44**	**Non-RhD antibodies, father neg.** **n = 30**	**Non-RhD-antibodies, father positive** **n = 45**

	**%**	**%**	**%**	**%**	**%**

***Perceived burden of the screening process 20th week of pregnancy***					

.no burden ^2^	99	52*	45*	60*	29*
.some burden	1	48	55	30	62
.significant burden	0	0	0	10	9

***Perceived burden of the screening process 2 weeks after birth***					

.no burden ^2^	99	67*	51*	67*	47*
.some burden	1	33	49	27	47
.significant burden	0	0	0	6	6

***Opinion on usefulness of the screening 2 weeks after birth***					

.not useful ^2^	8	19	22**	13	4
.some usefulness	60	43	45	57	56
.significant usefulness	32	38	33	30	40

***Opinion on balance usefulness versus burden of screening 2 weeks after birth***					

for myself					
. unimportant ^2^	7	0	9	4	4
. some importance	42	62	47	46	29
. important	51	38	44	50	67
for others					
. unimportant	5	5	12	4	4
. some importance	46	52	38	46	35
. important	49	43	50	50	61

^1 ^Weighted controls from hospital care: 35*0.41 = 15; 58 controls from midwifery care

^2 ^No burden/not useful/unimportant: VAS score < 3.4; some burden/usefulness/importance: VAS score between 3.4 and 6.9; significant burden/usefulness/important: VAS score >= 6.9 (see methods).

* p < 0.05, difference compared to controls **significant more 'no' than in control group (p 0.034)

All groups considered the perceived balance between usefulness and burden of the current screening program strongly in favour of antibody screening. In women at risk of HDFN (group E) the mean VAS score (not reported in Table 6) on this balance for themselves was higher than in the control group (p = 0.036), while the proportion of women with a score 'important' was slightly higher (no significance).

The multivariate analysis (Table 5) did not identify variables – in particular no risk or test relevant variables – explaining the overall attitude (from the perspective of other women) towards red cell antibody screening.

## Discussion

Our study shows that some psychological impact of pregnancy screening for non-RhD RBC antibodies exists in terms of anxiety, which is temporary and never unacceptable. Screen-positive women clearly experience a need for more information than provided, where universal preference for personal communication (rather than letters or e-mail) is stated. The overall attitude towards RBC antibody screening in pregnancy is strongly positive and does not depend on the screening result. Although screen-positive women experience some anxiety especially during the period of uncertainty about the actual HDFN risk status, they judge the balance between utility and burden of the screening process to be strongly positive. The anxiety level two weeks after birth does, reassuringly, not depend on the screening result or risk factors in this regard, but only on pre-existing background personal and clinical characteristics of the women.

Most literature about the attitude towards screening during pregnancy and the role of information concerns screening for maternal HIV status or for congenital malformations, conditions with a quite different impact on mother and child compared to the presence of non-RhD antibodies. Our results can best be compared with two prospective studies on screening for gestational diabetes [12,20]. Kerbel et al. show that positive-screened women feel more unsure shortly after screening; later in pregnancy there is no difference with negative-screened womenBoth studies on gestational diabetes screening show no differences in anxiety (STAI trait) and depression between women with a positive and a negative screen result. Unlike our results screen-positive and screen-negative women were satisfied about the explanation given about the screening test and the screening result. The difference may be explained by the complexity of non-RhD immunization with concomitant complex screening procedure compared to gestational diabetes, which is more easily explained. An alternative explanation is that well informed women are more aware of the complexity and the remaining gaps in the received information. Put to the extreme: the wish for more information indicates that information provision has been successful, rather than unsuccessful.

No previous studies offer explicit judgement on RhD or non-RhD RBC antibody screening, preventing comparison of our overall judgment results with others.

Our measurement design included asking women on experiences/attitudes in the recent past. This can cause some bias in the measurement of anxiety around the first and second blood test and during laboratory and clinical monitoring. Some research induced anxiety cannot be avoided, if so many questions are asked about anxiety during the different stages of the screening process. However, anxiety is measured in all groups according to the same procedure. Therefore, the differences and the impact of the various screening results can we interpreted in a meaningful way.

We did not include non-Dutch speaking women (± 5% of the population), so conclusions about this group cannot be drawn. Because there was no information about the proportion of eligible women without clinically relevant antibodies who actually participated in the study, we cannot be sure that our study population was representative for the whole population of Dutch-speaking pregnant women. However, in our multivariate analysis background characteristics did not emerge as significant predictors for information satisfaction, anxiety or overall attitude. Theoretically it is possible that this is due to selective non-response of women with specific background characteristics. However, it is unlikely that selective non-response will substantially change our conclusions.

In the Dutch setting of obstetric care provision of adequate explicit information on all standard screening tests during pregnancy is required (political imperative). In other countries standard screening tests may be performed without saying, avoiding some 'information induced' anxiety and curiosity.

## Conclusion

From our study it can be concluded that the adverse psychological effects of non RhD antibody screening, especially in women with a false positive test (not-confirmed positive screening or clinically non-relevant antibodies), are less important compared with the benefits in the small group of pregnancies with HDFN. Assuming that the non-RhD antibody screening program fulfils the other criteria of Jüngner and Wilson, our study primarily provides some issues for improvement:

- Written information should be distributed by obstetric care workers themselves, especially to screen-positive women;

- This information should describe the implications for mother and child of clinically relevant antibodies, but also of clinically non-relevant antibodies. Information about this last topic is very limited in the available booklets. It should be clear that in presence of clinically non-relevant antibodies there is no risk at all for mother and child;

- All important results, including a positive screen result, should be communicated personally to the pregnant woman;

- Theoretically one could consider pre test measurement of background anxiety (i.e. STAI) to select women who are more likely to develop high levels of anxiety if offered prenatal tests or during the test procedures. In view of increasing intensity of prenatal and even preconceptional screening programs, this consideration in our view is no longer theoretical given the strong effect of background anxiety and the possible predictive value for the course of pregnancy and delivery [21]. However, such an approach would also require obstetric care workers to be better equipped.

In conclusion we can say that the non-RhD antibody screening program is fully acceptable to pregnant women and fulfils the sixth criterium of Wilson and Jüngner,

## Competing interests

The authors declare that they have no competing interests.

## Authors' contributions

All authors participated in the study design, interpretation of the data and in writing of the paper. JK and TV participated in data collection. JK, TV and GB participated in data analysis. All authors have seen and approved the final version of the paper.

## Pre-publication history

The pre-publication history for this paper can be accessed here:



## Supplementary Material

Additional file 1 **Prenatal questionnaire women's attitude** Click here for file

Additional file 2 **Postnatal questionnaire cases at risk** Click here for file

Additional file 3 **Postnatal questionnaire women not at risk** Click here for file

## References

[B1] Committee prevention pregnancy immunisation (1992). Prevention of pregnancy immunisation.

[B2] Health Care Insurance Board (1998). Blood testing in pregnancy. Pregnancy immunisation, hepatitis B and lues. Health Care Insurance Board Inspection Public Health [Dutch].

[B3] Moise KJ (2000). Non-anti-D antibodies in red-cell alloimmunization. Eur J Obstet Gynecol Reprod Biol.

[B4] Moise KJ (2005). Red blood cell alloimmunization in pregnancy. Semin Hematol.

[B5] van Kamp IL, Klumper FJ, Oepkes D, Meerman RH, Scherjon SA, Vandenbussche FP (2005). Complications of intrauterine intravascular transfusion for fetal anemia due to maternal red-cell alloimmunization. Am J Obstet Gynecol.

[B6] Koelewijn JM, Vrijkotte TG, Schoot CE Van der, Bonsel GJ, de Haas M (2008). Effect of screening for red cell antibodies, other than anti-D, to detect hemolytic disease of the fetus and newborn: a population study in the Netherlands. Transfusion.

[B7] Urbaniak SJ, Greiss MA (2000). RhD: haemolytic disease of the fetus and the newborn. Blood Rev.

[B8] Wilson JMG, Jüngner G (1968). Principles and practice of screening for disease. Public Health Papers.

[B9] Stichting Perinatale Registratie Nederland (2007). Perinatal Care in the Netherlands 2004.

[B10] Oepkes D, Seaward PG, Vandenbussche FP, Windrim R, Kingdom J, Beyene J (2006). Doppler ultrasonography versus amniocentesis to predict fetal anemia. N Engl J Med.

[B11] Wildschut HI, Peters TJ, Weiner CP (2006). Screening in women's health, with emphasis on fetal Down's syndrome, breast cancer and osteoporosis. Hum Reprod Update.

[B12] Kerbel D, Glazier R, Holzapfel S, Yeung M, Lofsky S (1997). Adverse effects of screening for gestational diabetes: a prospective cohort study in Toronto, Canada. J Med Screen.

[B13] Rumbold AR, Crowther CA (2002). Women's experiences of being screened for gestational diabetes mellitus. Aust N Z J Obstet Gynaecol.

[B14] Fransen MP, Essink-Bot ML, Oenema A, Mackenbach JP, Steegers EA, Wildschut HI (2007). Ethnic differences in determinants of participation and non-participation in prenatal screening for Down syndrome: a theoretical framework. Prenat Diagn.

[B15] Wallston KA, Wallston BS, DeVellis R (2007). Development of the multidimensional health locus of control (MHLC) scales. Health Education Monographs.

[B16] Spielberger CD, Gorusch RL, Lushene LE, Palo Alto C (1970). STAI manual for the state-trait anxiety inventory.

[B17] Spielberger CD, Pao Alto C (1983). State-trait anxiety inventory for adults. Sampler set, manual, test, scoring key.

[B18] Krabbe PF, Salomon JA, Murray CJ (2007). Quantification of health states with rank-based nonmetric multidimensional scaling. Med Decis Making.

[B19] Balon R (2007). Rating scales for anxiety/anxiety disorders. Curr Psychiatry Rep.

[B20] Rumbold AR, Crowther CA (2002). Women's experiences of being screened for gestational diabetes mellitus. Aust N Z J Obstet Gynaecol.

[B21] Wijnen H (2005). The Kempen study: Aspects of maternal well-being and obstetrical outcome in relation to gestational thyroid function. Thesis.

