# Supplementary material for: Women's attitude towards prenatal screening for red blood cell antibodies, other than RhD
Source: BMC Pregnancy Childbirth. 2008 Nov 11;8:49. doi: 10.1186/1471-2393-8-49 (PMC2605433; doi:10.1186/1471-2393-8-49)
Supplement: Additional file 1 — Prenatal questionnaire women's attitude [file 1471-2393-8-49-S1.doc]

**Pp-nummer:     **

VRAGENLIJST

**voor zwangere vrouwen**

onderzoek naar bloedgroepantistoffen in de zwangerschap


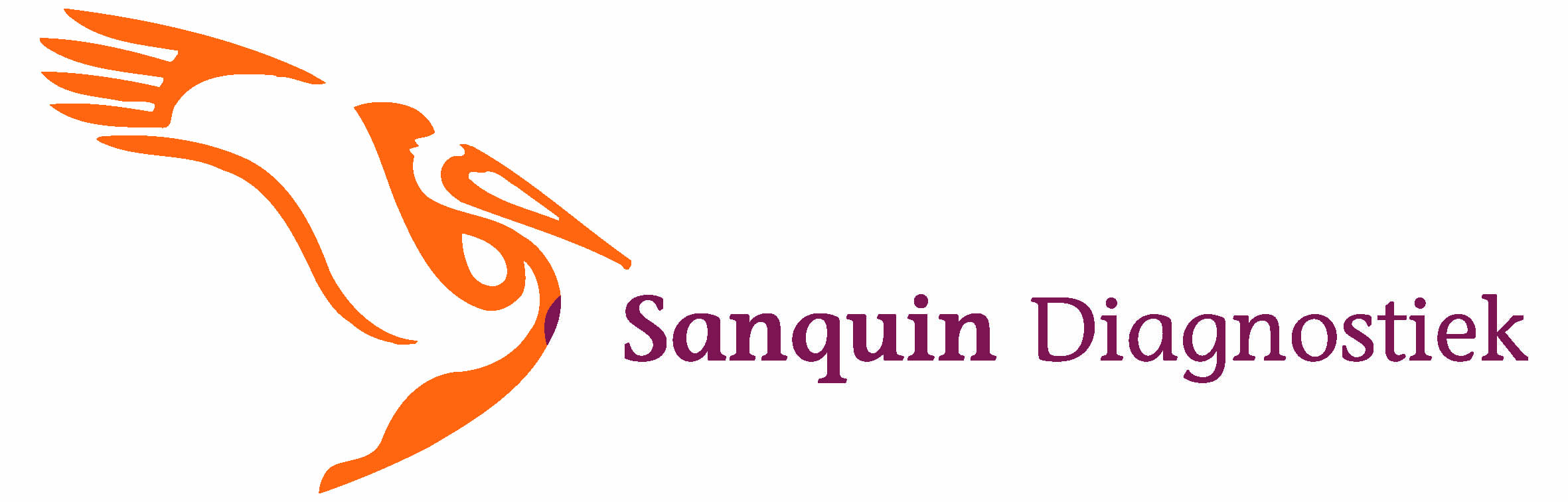


**Sanquin-CLB, OPZI-onderzoek, Antwoordnummer 814 1000 SE AMSTERDAM telefoonnummer: 020- 5123452 (Joke Koelewijn of Rina Siemons)**

**AANWIJZINGEN VOOR HET INVULLEN**

Beantwoord de vragen van deze vragenlijst in de volgorde waarin ze gesteld zijn. De vragenlijst bestaat uit 10 ‘blokken’ met in totaal 131 vragen.

**INVULLEN:**

- U Kruist bij elke vraag **één antwoord** aan, behalve wanneer duidelijk is aangegeven dat u meerdere antwoorden kunt aankruisen.
- Denk niet te lang na over een antwoord. Vul het antwoord in dat u het eerste in gedachten komt. Het gaat om uw mening; **elk antwoord is goed**.
- Bij sommige antwoorden is aangegeven dat u na dit antwoord een aantal vragen kunt overslaan en meteen door kunt gaan naar een andere vraag. Wanneer er bijvoorbeeld staat dat u door kunt gaan naar vraag **8**, dan heeft dit vraagnummer een kleur gekregen, zodat u meteen kunt zien waar – in dit geval –vraag **8.** staat.

**FORMULIER PERSOONSGEGEVENS:**

- Vul formulier P. in. We vragen u hierop naar uw naam, adres en telefoonnummer. Uw adres hebben we nodig om u de tweede vragenlijst én - als dank voor uw medewerking - een aardigheidje toe te kunnen sturen. Uw telefoonnummer willen we graag hebben om u zo nodig te kunnen bellen als de antwoorden op bepaalde vragen niet duidelijk zijn. Deze persoonlijke gegevens worden vernietigd zodra we beide vragenlijsten compleet binnen hebben gekregen.
- Mocht u 10 dagen na de bevalling de tweede vragenlijst nog niet hebben ontvangen, dan stellen we het op prijs als u ons dit laat weten. We sturen u dan alsnog de lijst toe. Adres/telefoonnummer:

Sanquin, CLB,

OPZI, postvak 40,

antwoordnummer 814,

1000 SE Amsterdam (een postzegel is niet nodig).

[opzi@amc.uva.nl](mailto:opzi@amc.uva.nl)

020-5123452

| 1. Algemeen |
| --- |

**1. Wat is uw leeftijd?**  jaar

**2. Wat zijn de 4 cijfers van uw postcode?** 

## (Dit willen wij weten omdat het bloedonderzoek op antistoffen niet in

## elke regio op dezelfde wijze wordt uitgevoerd.)

**3. Wat is de hoogste opleiding die u hebt voltooid?**

□ Hoger Beroepsonderwijs/ Universitair onderwijs

□ Middelbaar Beroepsonderwijs/ HAVO/ VWO

□ Lager Beroepsonderwijs/ MAVO/ VMBO

□ Lager onderwijs

□ Geen

**4. Wat is uw beroep? Vul deze vraag ook in als u op dit moment niet werkt.**

…………..……………..…………………………..

**5.** **Hebt u een partner?**

□ ja, wij wonen samen □ ja, wij wonen niet samen □ nee

in één huishouden

**6.** **Wat is het beroep van uw partner?** **Vul deze vraag ook in als**

**uw partner op dit moment niet werkt.**

…………..……………..…………………………..

**7.** **Wat is de hoogste opleiding die uw partner heeft voltooid?**

□ Hoger Beroepsonderwijs/Universitair onderwijs

□ Middelbaar Beroepsonderwijs, HAVO, VWO

□ Lager Beroepsonderwijs, MAVO, VMBO

□ Lager onderwijs

□ Geen

*ga verder naar vraag* **8.**

Bepaalde antistoffen komen vaker voor óf komen juist helemaal niet voor bij vrouwen uit verschillende landen en regio’s. Daarom willen we graag weten waar u geboren bent en tot welke bevolkingsgroep u behoort.

**8. In welk land zijn u en uw ouders en uw eventuele partner en (schoon)ouders**

**geboren?**

| geboorteland van uzelf |  | geboorteland van uw partner |  |
| --- | --- | --- | --- |
| geboorteland van uw vader |  | geboorteland vader van uw partner |  |
| geboorteland van uw moeder |  | geboorteland moeder van uw partner |  |

1. **Tot welke bevolkingsgroep rekenen u en uw eventuele partner zichzelf?**

**uzelf uw partner**

**Nederlands**  □ □

**Marokkaans** □ □

**Turks** □ □

**Andere landen rond de Middellandse Zee** □ □

**Ander Europees**  □ □

(andere Europese landen, VS, Canada)

**Creools** (Afrikaans, Surinaams/Antilliaans □ □

van negroïde afkomst)

**Hindoestaans** (Surinaams-Hindoestaans, □ □

Indiaas, Pakistaans)

**Aziatisch** (Chinees, Japans, Indonesisch, □ □

Ambonees, Vietnamees)

**Overige** (o.a. Zuid-Amerikaans) □ □

| 2. Deze zwangerschap |
| --- |

**10. Wat is uw uitgerekende datum?** ____

d d m m j j j j

**11. Door wie wordt uw zwangerschap op dit moment gecontroleerd?**

□ verloskundige

□ gynaecoloog

□ huisarts

12. Vindt u dat het op dit moment goed gaat met u en met uw zwangerschap?

□ ja nee, het gaat niet goed met: □ mijzelf

□ mijn kind

□ mijn kind en mijzelf

Als het niet goed gaat, wilt u dit hieronder voor ons toelichten? We hopen dat u toch bereid bent om ook de rest van de vragen zoveel mogelijk te beantwoorden.

…………………………………………………………………………………………………

…………………………………………………………………………………………………

…………………………………………………………………………………………………

**13. Waar bent u van plan te gaan bevallen?**

□ waarschijnlijk thuis

□ waarschijnlijk in het ziekenhuis

□ waarschijnlijk in een kraamkliniek/kraamhotel

## □ dat weet ik nog niet

| 3. Eerdere zwangerschappen |
| --- |

**14. Bent u eerder zwanger geweest of bevallen?**

Hierbij tellen ook miskramen en abortussen mee.

□ ja □ nee

*ga verder naar vraag* ***19.***

**15. Hebt u weleens een miskraam of een abortus gehad?**

□ ja □ nee

………… miskramen/abortussen

**16. Hoe vaak bent u eerder bevallen?**

Wij spreken pas van een bevalling als het kindje na 16 weken geboren is.

Ook een eventueel doodgeboren kindje telt mee. Een bevalling van een tweeling telt als één bevalling.

………… keer bevallen

1. **Zijn er naar uw gevoel problemen geweest tijdens één of meer eerdere**

**zwangerschappen?**

□ nee ja, er waren problemen met □ mijzelf

□ mijn kind

□ mijn kind en mijzelf

Zo ja, wilt u hieronder deze problemen kort beschrijven?

…………………………………………………………………………………………………

…………………………………………………………………………………………………

…………………………………………………………………………………………………

18. Bent u in het verleden wel eens geconfronteerd met een afwijkende

testuitslag tijdens of kort na een zwangerschap?

□ nee □ ja

Zo ja, om welke testuitslag ging het en hoe liep het verder af?

……………………………………………………………..……………………………………

……………………………………………………………..……………………………………

| 4. Bloedgroep-antistoffen |
| --- |

Bij de eerste zwangerschapscontrole wordt bloed afgenomen. Sinds 1 juli 1998 wordt dit bloed altijd onderzocht op de aanwezigheid van antistoffen tegen rode bloedcellen.

19. Wist u – voordat u deze vragenlijst kreeg – dat uw bloed wordt onderzocht op

antistoffen tegen rode bloedcellen?

□ Ja, dat wist ik

□ Ik had er, geloof ik, wel iets over gehoord

□ Nee, dat wist ik niet

20. Hebt u wel eens meegemaakt dat een zwangere in uw omgeving antistoffen

tegen rode bloedcellen in haar bloed had, bijvoorbeeld rhesusantistoffen of

andere bloedgroep-antistoffen?

□ Nee

□ Ik geloof van wel

□ Ja

21. Hebt u misschien in uw omgeving wel eens meegemaakt dat een ongeboren

of pasgeboren kind ziek werd door bloedgroep-antistoffen?

□ Nee

□ Ik geloof van wel

□ Ja

22. Hebt u uw uitslag van het onderzoek naar bloedgroep-antistoffen in deze

zwangerschap gehoord?

□ Ja □ Ik geloof van wel □ Nee

*ga verder naar vraag* ***27.***

**23. Op welke manier hebt u uw uitslag gehoord?**

□ op het spreekuur □ telefonisch □ per brief

□ anders, namelijk ……………………………………………………………………………

24. Deze uitslag was:

□ Afwijkend (niet in orde) □ Niet afwijkend (in orde)

□ de test was aanvankelijk afwijkend, maar bleek

bij verder onderzoek toch goed te zijn *ga verder naar vraag* ***27.***

□ er zijn wel antistoffen gevonden, maar deze kunnen voor de baby geen kwaad

□ er zijn antistoffen gevonden die mogelijk kwaad kunnen voor de baby

25. Wist u al vóór deze zwangerschap dat u antistoffen in uw bloed hebt?

□ Nee

□ Ik geloof van wel

□ Ja

**26. Is het bloed van uw partner onderzocht?**

□ ja, in deze □ ja, in een eerdere □ nee **□** ik weet het niet

zwangerschap zwangerschap

*ga verder naar vraag* ***27.27.***

De uitslag van het bloedonderzoek van mijn partner is:

□ mijn partner heeft de bloedgroep niet waartegen ik antistoffen heb; verdere controle

in de zwangerschap is niet nodig

□ mijn partner heeft de bloedgroep wel waartegen ik antistoffen heb; mijn bloed wordt

verder nog gecontroleerd op bloedgroep-antistoffen

□ (nog) niet bekend

| 5. Informatie over bloedgroep-antistoffen |
| --- |

# De volgende vragen betreffen de informatie die u gekregen hebt of zelf gevonden hebt over het onderzoek op bloedgroep-antistoffen.

# Bij de eerste zwangerschapscontrole wordt soms de folder **Zwanger!** uitgedeeld, waarin o.a. beschreven staat welke bloedonderzoeken er in de zwangerschap verricht worden.

**27. De folder Zwanger!**

□ heb ik nooit gezien *ga verder naar vraag* ***30.***

□ heb ik wel gekregen, maar nog niet gelezen

□ heb ik even doorgekeken

□ heb ik goed gelezen

1. Hebt u in de folder Zwanger! iets over bloedgroep-antistoffen gelezen?

□ Ja □ Ik geloof van wel □ Nee

29. Hoe kwam u aan de folder Zwanger! ?

□ ik heb deze gekregen van mijn verloskundige

□ ik heb deze gekregen van mijn gynaecoloog

□ ik heb deze gekregen van mijn huisarts

□ ik heb deze gekregen via Internet

□ ik heb deze gekregen van een kennis

□ anders, namelijk ………………………………………………….………………………

1. Hebt u nog andere folders gelezen met informatie over het bloedonderzoek

op bloedgroep-antistoffen?

□ Ja □ Ik geloof van wel □ Nee

*ga verder naar vraag* ***33.***

**31. Welke folders zijn dit?** …………… …………………………………………………..

……………………………………………………………………………………………

32. Hoe kwam u aan deze folder(s)?

□ ik heb deze gekregen van mijn verloskundige

□ ik heb deze gekregen van mijn gynaecoloog

□ ik heb deze gekregen van mijn huisarts

□ ik heb deze gekregen via Internet

□ ik heb deze gekregen van een kennis

□ anders, namelijk ………………………………………………….………………………

33. Hebt u van uw zorgverlener mondeling uitleg gekregen over het

bloedonderzoek op bloedgroep-antistoffen?

□ Ja □ Ik geloof van wel □ Nee

34. Hebt u nog op andere wijze iets gehoord of gelezen over het bloedonderzoek

op antistoffen?

U kunt meerdere zaken aankruisen

□ tijdschrift(en), krant(en)

□ boek(en)

□ Internet, site ………………………………………………………………..

□ televisie of radio

□ via bekenden

□ vanuit mijn opleiding of beroep

□ anders, namelijk ………………………………………………………………………...….

| 6. Mening over de informatie |
| --- |

*Hiervoor ging het erom welke informatie u gehoord en gelezen hebt.*

*In dit onderdeel van de vragenlijst vragen we of u deze informatie duidelijk vindt en voldoende. Dit willen we weten om in de toekomst de informatie af te kunnen stemmen op wat bij zwangeren zelf als behoefte leeft.*

Bij de volgende vragen is het de bedoeling dat u uw antwoord aangeeft op een schaal. U kunt dat doen door een kruisje te zetten op de lijn op het punt dat het beste met uw mening overeenkomt.

**Bijvoorbeeld:**

**De informatie over de bereikbaarheid van mijn verloskundige zorgverlener in het weekend vind ik:**

heel onduidelijk onduidelijk duidelijk zeer duidelijk

*Dit kruisje geeft aan dat u de informatie behoorlijk duidelijk vindt.*

***Hierna volgen de echte vragen.***

## Over de hoeveelheid schriftelijke informatie over bloedgroep-antistoffen die ik heb gekregen via mijn verloskundige zorgverlener, ben ik:

zeer ontevreden ontevreden tevreden zeer tevreden

Als u géén folder(s) over bloedgroep-antistoffen gekregen of zelf gevonden hebt, sla dan vraag 36 over en ga door naar vraag 37.

1. **De folder(s) die ik heb gelezen over het onderzoek op bloedgroep-antistoffen vind ik:**

heel onduidelijk onduidelijk duidelijk zeer duidelijk

1. Over de hoeveelheid mondelinge informatie van mijn verloskundige zorgverlener over bloedgroep-antistoffen ben ik:

zeer ontevreden ontevreden tevreden zeer tevreden

1. **De mondelinge informatie van mijn verloskundige zorgverlener over bloedgroep-antistoffen vind ik:**

heel onduidelijk onduidelijk duidelijk zeer duidelijk

1. **Over de hoeveelheid schriftelijke informatie die ik zelf heb kunnen vinden over bloedgroep- antistoffen ben ik:**

zeer ontevreden ontevreden tevreden zeer tevreden

Als u zelf geen schriftelijke informatie over bloedgroep-antistoffen hebt gezocht of deze niet hebt gevonden, sla dan vraag 40 over en ga door naar vraag 41.

1. **De schriftelijke informatie over bloedgroepantistoffen die ik zelf gevonden heb vind ik:**

heel onduidelijk onduidelijk duidelijk zeer duidelijk

1. Had u in uw geval nog meer informatie willen krijgen over bloedgroep-antistoffen?

□ Nee ………………………………………………………………………………………

□ Ja:

U kunt meerdere zaken aankruisen:

□ meer schriftelijke informatie

□ meer mondelinge informatie van mijn verloskundige zorgverlener

□ via een speciaal telefoonnummer

□ anders ……………………………………………………………………

| 7. Kennis over bloedgroep-antistoffen |
| --- |

## *In de volgende vragen kijken we wat u op dit moment weet over bloedgroep-antistoffen. Als u het antwoord niet weet, geeft u dit dan gewoon aan, u hoeft immers niet alles te weten.*

## *Het is niet de bedoeling dat u naar informatie gaat zoeken om het juiste antwoord op de vraag te vinden. We willen namelijk weten waar we bij voorlichting vanuit moeten gaan.*

1. **In iedere zwangerschap worden de bloedgroep en de rhesusfactor bepaald,**

**bijvoorbeeld A pos of A neg.**

□JA □ NEE □ ik weet het niet

1. Alleen de moeder bepaalt de bloedgroep van het kind.

□JA □ NEE □ ik weet het niet

44. Rhesus-antistoffen kunnen gevaarlijk zijn voor het kind.

□JA □ NEE □ ik weet het niet

1. **Bij mogelijk gevaarlijke bloedgroep-antistoffen wordt ook de bloedgroep van de biologische vader bepaald.**

□JA □ NEE □ ik weet het niet

1. **Mogelijk gevaarlijke bloedgroep-antistoffen komen bij zwangeren in Nederland voor bij:**

Geef aan welk antwoord volgens u het meest juist is.

□ 1 op de 10 zwangeren

□ 1 op de 30 zwangeren

□ 1 op de 100 zwangeren

□ 1 op de 300 zwangeren

□ 1 op de 1000 zwangeren

□ 1 op de 3000 zwangeren

1. **Er zijn verschillende soorten bloedgroep-antistoffen, die allemaal gevaarlijk**

**zijn voor het kind.**

□JA □ NEE □ ik weet het niet

1. **Als de moeder bloedgroep-antistoffen heeft, dan zal haar dochter als ze ooit zwanger wordt, ook bloedgroep-antistoffen in haar bloed hebben.**

□JA □ NEE □ ik weet het niet

1. **Als er in het bloed van de zwangere bloedgroep-antistoffen aanwezig zijn, dan kunnen de gevolgen voor moeder en kind zijn:**

# Kind

1. Bloedarmoede □JA □ NEE □ ik weet het niet

2. Allergie □JA □ NEE □ ik weet het niet

3. Geelzucht □JA □ NEE □ ik weet het niet

4. Darmkrampjes □JA □ NEE □ ik weet het niet

##### Moeder

1. Bloedarmoede □JA □ NEE □ ik weet het niet

2. Misselijkheid □JA □ NEE □ ik weet het niet

3. Veel bloedverlies □JA □ NEE □ ik weet het niet

na de bevalling

4. Borstontsteking □JA □ NEE □ ik weet het niet

1. **Als er sprake is van ernstige bloedafbraak bij het kind, dan kan men het kind in de buik van de moeder een bloedtransfusie geven.**

□JA □ NEE □ ik weet het niet

1. **Onbehandelde ernstige geelzucht door bloedafbraak kan hersenschade veroorzaken bij de pasgeborene.**

□JA □ NEE □ ik weet het niet

| 8. Ervaringen met het bloedonderzoek |
| --- |

Het laatste onderdeel van deze vragenlijst gaat over de vraag in hoeverre (de uitslag van) het bloedonderzoek naar bloedgroep-antistoffen u bezig heeft gehouden.

LET OP:

Deze vragen gaan niet over andere bepalingen in het bloed zoals HIV, hepatitis en dergelijke, maar uitsluitend over bloedgroep-antistoffen.

Nadat bij u voor de eerste keer tijdens deze zwangerschap bloed is afgenomen voor onderzoek op o.a. bloedgroep-antistoffen, moest u een poosje wachten op de uitslag.

1. **Hebt u in die wachtperiode op de uitslag van het eerste bloedonderzoek tijdens deze zwangerschap nagedacht over de uitslag van het bloedonderzoek naar bloedgroep-antistoffen?**

nooit af en toe bijna elke dag meerdere keren

per dag

1. **Bent u in de tijd dat u op de uitslag van het eerste bloedonderzoek wachtte ongerust geweest dat de uitslag van het bloedonderzoek niet goed zou zijn?**

helemaal niet een beetje nogal heel erg

1. **In vraag 23 hebt u aangegeven op welke manier u de uitslag heeft gekregen.**

**Op welke manier had u bij voorkeur deze uitslag gekregen?**

□ op het spreekuur □ telefonisch □ per brief

□ anders, namelijk ……………………………………………………………………………

Bij onderdeel 4, vraag 24, hebt u al ingevuld wat de testuitslag was.

□ niet afwijkend (in orde) □ afwijkend (mogelijk niet in orde)

*ga verder naar vraag* ***55.*** *ga verder naar vraag* ***56.***

Toen u gehoord had dat de uitslag goed was, kan dat voor u vanzelfsprekend geweest zijn, maar mogelijk voelde u zich ook wel opgelucht.

1. Geef aan in hoeverre er bij u sprake was van opluchting na de goede

testuitslag.

helemaal niet een beetje nogal heel erg

*U kunt meteen verder gaan naar vraag* ***68****.*

56. Bent u geschrokken van de afwijkende testuitslag?

helemaal niet een beetje nogal heel erg

# Soms kreeg u meteen de uitslag, zoals u die bij vraag 24 hebt ingevuld. Het kan ook zijn dat er eerst nog voor een tweede keer bloed is afgenomen voordat u de definitieve uitslag kreeg.

1. Is er bij u nog een tweede keer bloed afgenomen om definitief te bepalen om

welke soort bloedgroep-antistoffen het gaat?

□ ja, er is opnieuw bloed afgenomen □ nee

*ga verder naar vraag* ***59.***

Ook nu moest u een poosje wachten op de uitslag van dit tweede bloedonderzoek.

# **Hebt u in de wachttijd tot de uitslag nagedacht over de uitslag van dit**

# **tweede bloedonderzoek?**

nooit af en toe bijna elke dag meerdere keren

per dag

1. **Bent u in de periode dat u moest wachten op de uitslag van het tweede bloedonderzoek ongerust geweest dat deze opnieuw niet goed zou zijn?**

helemaal niet een beetje nogal heel erg

1. **Uit het onderzoek kan gebleken zijn dat er wel of niet antistoffen gevonden**

**zijn die voor de baby mogelijk gevaarlijk zijn. In vraag 24 hebt u dat ook al**

**ingevuld.**

□ geen gevaarlijke antistoffen □ antistoffen die mogelijk

gevaarlijk zijn voor de baby

*ga verder naar vraag* ***60.*** *ga verder naar vraag* ***62.***

1. **Geef aan in hoeverre u opgelucht was toen bleek dat er geen antistoffen zijn**

**gevonden die gevaarlijk kunnen zijn voor de baby.**

helemaal niet een beetje nogal heel erg

**61. Geef aan in hoeverre u gerustgesteld was toen bleek dat er geen antistoffen**

**zijn gevonden die gevaarlijk kunnen zijn voor de baby.**

helemaal niet niet helemaal redelijk volledig

*U kunt nu verder gaan naar vraag* ***68.***

**62. Bent u geschrokken van de uitslag?**

helemaal niet een beetje nogal heel erg

Hierna is vermoedelijk het bloed van uw partner onderzocht om te kijken of de bloedgroep waartegen u antistoffen hebt bij hem aanwezig is.

□ partner onderzocht □ partner niet onderzocht

*ga verder naar vraag* ***63.*** *ga verder naar vraag* ***68.***

Ook nu moest u een poosje wachten op de uitslag van het bloedonderzoek van uw partner.

# **63. Hebt u in die periode nagedacht over de uitslag van het bloedonderzoek van**

# **uw partner?**

nooit af en toe bijna elke dag meerdere keren

per dag

1. **Bent u in de periode dat u moest wachten op de uitslag van het bloedonderzoek van uw partner ongerust geweest over de uitslag?**

helemaal niet een beetje nogal heel erg

De uitslag van het bloedonderzoek van uw partner kan zijn dat hij de bloedgroep waartegen u antistoffen hebt wel of niet heeft. U hebt dat ingevuld bij vraag 26.

Als hij deze bloedgroep niet heeft, wordt er verder geen bloed meer bij u gecontroleerd. Als hij deze bloedgroep wel heeft, wordt uw bloed nog regelmatig gecontroleerd in de zwangerschap.

□ partner heeft bloedgroep niet □ partner heeft bloedgroep wel

*ga verder naar vraag* ***67.***

**65. Geef aan in hoeverre u opgelucht was toen uw partner de bloedgroep**

**waartegen u antistoffen hebt, niet bleek te hebben.**

helemaal niet een beetje nogal heel erg

1. **Geef aan in hoeverre u gerustgesteld was toen uw partner de bloedgroep waartegen u antistoffen hebt, niet bleek te hebben.**

helemaal niet niet helemaal redelijk volledig

*U kunt nu meteen verder gaan naar vraag 68.*

Bij de uitslag bleek uw partner de bloedgroep waartegen u antistoffen hebt, wel te hebben. U wordt daarom nog regelmatig gecontroleerd tijdens de zwangerschap.

**67. Bent u geschrokken van de uitslag van het bloedonderzoek van uw partner?**

helemaal niet een beetje nogal heel erg

68. Geef aan of u zich op dit moment zorgen maakt over bloedgroep-antistoffen

en de gevolgen daarvan voor de zwangerschap.

helemaal niet een beetje nogal heel erg

**69. Geef aan of u zich op dit moment zorgen maakt over de gezondheid van uw**

**kind.**

helemaal niet een beetje nogal heel erg

**70. Hebben eventuele zorgen over uw zwangerschap geleid tot veranderingen in**

**uw leefwijze, zoals:**

U kunt meerdere zaken aankruisen

□ meer roken □ ongezonder eten

□ meer alcoholgebruik □ slechter slapen

□ medicijngebruik □ anders…….. ……………………

Als afsluiting van dit onderdeel stellen we nog 3 vragen waarin uw totale oordeel kunt geven over het bloedonderzoek op bloedgroep-antistoffen.

1. **Geef aan in hoeverre het onderzoek op bloedgroep-antistoffen en eventuele vervolgonderzoeken voor uzelf psychisch belastend is geweest tot nu toe.**

helemaal niet een beetje nogal heel erg

1. **Geef aan in hoeverre u het onderzoek op bloedgroep-antistoffen en eventuele vervolgonderzoeken psychisch belastend zou vinden voor een andere zwangere, bijvoorbeeld wanneer een goede vriendin u hiernaar zou vragen.**

helemaal niet een beetje nogal heel erg

1. Zijn er nog zaken over het onderzoek naar bloedgroep-antistoffen die niet aan de orde zijn geweest? Hebt u tips om het bloedonderzoek te verbeteren?

……………………………………………………………………………………………………..

……………………………………………………………………………………………………..

| 9. Opvattingen over gezondheid |
| --- |

*Sommige mensen maken zich over hun gezondheid helemaal geen zorgen, andere mensen twijfelen daaraan. De volgende vragen zijn bedoeld om te meten hoe u denkt over uw gezondheid en wat u daar zelf aan kunt doen.*

*Hieronder volgt een lijst met een aantal uitspraken over gezondheid en ziekte. Geef voor elke uitspraak aan in welke mate u het hiermee eens bent of niet. U kunt dit doen door het hokje onder het antwoord dat voor u van toepassing is in te vullen.*

*Denkt u bij het invullen niet te lang na: het gaat vooral om uw eerste reactie.*

beslist mee enigszins enigszins mee beslist

mee eens eens mee eens mee oneens oneens mee oneens

74. Wat ik ook doe, ziek word ik toch.

75. Als ik ziek ben ligt het aan mijzelf

hoe snel ik beter word.

76. Mijn goede gezondheid is grotendeels

een kwestie van aanleg of geluk.

77. Als ik van een ziekte herstel, heb ik

dit toch vooral te danken aan mijn arts.

78. Als ik me niet goed voel, moet ik

eigenlijk een arts raadplegen.

79. Als ik goed voor mijzelf zorg, kan ik

ziekten voorkomen.

80. Wat betreft mijn gezondheid kan ik

alleen maar doen wat de dokter zegt.

81. Als ik ziek word dan is dat mijn eigen

schuld.

82. Artsen hebben er veel invloed op of ik

gezond ben of niet.

83. Hoe snel ik van een ziekte zal genezen

wordt grotendeels door geluk bepaald.

84.Hoe snel ik van een ziekte zal genezen

wordt grotendeels door geluk bepaald.

beslist mee enigszins enigszins mee beslist

mee eens eens mee eens mee oneens oneens mee oneens

84. Mijn gezondheid wordt in de eerste

plaats bepaald door wat ik zelf doe.

85. De meeste dingen waardoor ik ziek

wordt overkomen mij bij toeval.

86. Artsen bepalen mijn gezondheid.

87. Ik heb mijn gezondheid in eigen hand.

88. De beste manier voor mij om ziekten

te voorkomen is regelmatig de

huisarts raadplegen.

89. Of Ik gezond ben is een kwestie van

toevallige gebeurtenissen.

90. Het ligt vooral aan mijzelf hoe snel ik

van een ziekte zal genezen.

91. Als ik ziek word, dan word ik het toch,

daar kan niemand iets aan doen.

beslist mee enigszins enigszins mee beslist

mee eens eens mee eens mee oneens oneens mee oneens

beslist mee enigszins enigszins mee beslist

mee eens eens mee eens mee oneens oneens mee oneens

| 10. Zelf beschrijving |
| --- |

*Hieronder vindt u een aantal uitspraken, die mensen hebben gebruikt om zichzelf te beschrijven. Lees iedere uitspraak door en zet dan een kringetje om het cijfer rechts van die uitspraak om aan te geven hoe u zich nu voelt, dus nu op dit moment. Er zijn geen goede of slechte antwoorden. Denk niet te lang na en geef uw eerste indruk, die is meestal de beste. Het gaat er dus om dat u weergeeft wat u op dit moment voelt.*

geheel een tamelijk zeer

niet beetje veel veel

92. Ik voel me kalm . . . . . . . . . . . . . . . . . 1 2 3 4

93. Ik voel me veilig . . . . . . . . . . . . . . . . . 1 2 3 4

94. Ik ben gespannen . . . . . . . . . . . . . . . . . 1 2 3 4

95. Ik voel me onrustig . . . . . . . . . . . . . . . . 1 2 3 4

96. Ik voel me op mijn gemak . . . .. . . . . . . . . 1 2 3 4

97. Ik ben in de war. . . . . . . . . . . . . . . . . . 1 2 3 4

98. Ik pieker over nare dingen die kunnen gebeuren 1 2 3 4

99. Ik voel me voldaan . . . . . . . . . . . . . . . . 1 2 3 4

100. Ik ben bang . . . . . . . . . . . . . . . . . . . 1 2 3 4

101. Ik voel me aangenaam. . . . . . . . . . . . . . 1 2 3 4

102. Ik voel me zeker . . . . . . . . . . . . . . . . . 1 2 3 4

103. Ik voel me nerveus . . . . . . . . . . . . . . . . 1 2 3 4

104. Ik ben zenuwachtig . . . . . . . . . . . . . . . 1 2 3 4

105. Ik ben besluiteloos . . . . . . . . . . . . . . . . 1 2 3 4

106. Ik ben ontspannen . . . . . . . . . . . . . . . . 1 2 3 4

107. Ik voel me tevreden . . . . . . . . . . . . . . . 1 2 3 4

108. Ik maak me zorgen . . . . . . . . . . . . . . . . 1 2 3 4

109. Ik voel me gejaagd . . . . . . . . . . . . . . . . 1 2 3 4

110. Ik voel me evenwichtig . . . . . . . . . . . . . . 1 2 3 4

111. Ik voel me prettig . . . . . . . . . . . . . . . . . 1 2 3 4

geheel een tamelijk zeer

niet beetje veel veel

*Hieronder vindt u een aantal uitspraken, die mensen hebben gebruikt om zichzelf te beschrijven. Lees iedere uitspraak door en zet dan een kringetje om het cijfer rechts van die uitspraak om aan te geven hoe u zich in het algemeen voelt. Er zijn geen goede of slechte antwoorden. Denk niet te lang na en geef uw eerste indruk. Het gaat er dus om dat u bij deze vragenlijst weergeeft hoe u zich in het algemeen voelt.*

bijna soms vaak bijna

nooit altijd

112. Ik voel me prettig . . . . . . . . . . . . . . . . . . . 1 2 3 4

113. Ik voel me nerveus en onrustig . . . . . . . . . . . 1 2 3 4

114. Ik voel me tevreden . . . . . . . . . . . . . . . . . . 1 2 3 4

115. Ik kan een tegenslag maar heel moeilijk verwerken . 1 2 3 4

116. Ik voel me in vrijwel alles tekort schieten. . . . . . . 1 2 3 4

117. Ik voel me uitgerust . . . . . . . . . . . . . . . . . . 1 2 3 4

118. Ik voel me rustig en beheerst . . . . . . . . . . . . . 1 2 3 4

119. Ik voel dat de moeilijkheden zich opstapelen zodat

ik er niet meer tegenop kan . . . . . . . . . . . . . . 1 2 3 4

120. Ik pieker teveel over dingen die niet zo belangrijk zijn. 1 2 3 4

121. Ik ben gelukkig. . . . . . . . . . . . . . . . . . . . 1 2 3 4

122. Ik word geplaagd door storende gedachten. . . . . . 1 2 3 4

123. Ik heb gebrek aan zelfvertrouwen . . . . . . . . . . . 1 2 3 4

124. Ik voel me veilig . . . . . . . . . . . . . . . . . . . 1 2 3 4

125. Ik voel me op mijn gemak . . . . . . . . . . . . . . 1 2 3 4

126. Ik ben gelijkmatig van stemming . . . . . . . . . . 1 2 3 4

127. Ik ben tevreden . . . . . . . . . . . . . . . . . . . 1 2 3 4

128. Er zijn gedachten die ik heel moeilijk los kan laten. . 1 2 3 4

129. Ik neem teleurstellingen zo zwaar op dat ik ze niet van

me af kan zetten . . . . . . . . . . . . . . . . . . . 1 2 3 4

130. Ik ben een rustig iemand . . . . . . . . . . . . . . 1 2 3 4

131. Ik raak helemaal gespannen en in beroering als ik

denk aan mijn zorgen van de laatste tijd . . . . . . . 1 2 3 4

bijna soms vaak bijna

nooit altijd

**Z.O.Z.**

U bent nu klaar met het invullen van deze vragenlijst.

Wilt u deze direct naar ons terugsturen in de bijgevoegde antwoordenvelop?

Vergeet ook niet formulier P mee te sturen.

Hartelijk dank voor de medewerking.

Als u geen antwoordenvelop heeft, kunt u de vragenlijst terugsturen naar:

Sanquin, CLB

OPZI-onderzoek, postvak 40

Antwoordnummer 814

1000 SE AMSTERDAM

Hier hoeft geen postzegel op.

Ook kunt u ons bellen, zodat wij u alsnog een antwoordenvelop kunnen toesturen:

020- 5123452 (Joke Koelewijn of Rina Siemons)

Rond uw uitgerekende datum sturen wij u de tweede vragenlijst toe, die u twee weken na de bevalling kunt invullen.
