# Supplementary material for: Women's attitude towards prenatal screening for red blood cell antibodies, other than RhD
Source: BMC Pregnancy Childbirth. 2008 Nov 11;8:49. doi: 10.1186/1471-2393-8-49 (PMC2605433; doi:10.1186/1471-2393-8-49)
Supplement: Additional file 3 — Postnatal questionnaire women not at risk [file 1471-2393-8-49-S3.doc]

**CO Pp-nummer:      **

VRAGENLIJST

**voor vrouwen**

**twee weken na de bevalling**

onderzoek naar bloedgroepantistoffen in de zwangerschap


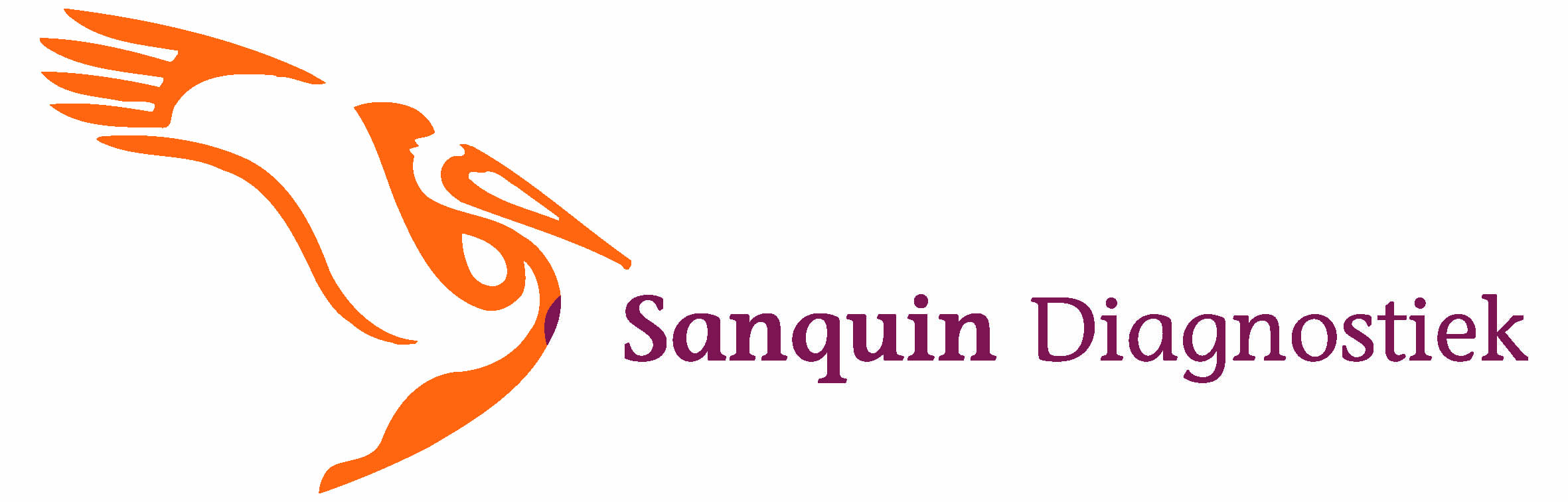


**AANWIJZINGEN VOOR HET INVULLEN**

Beantwoord de vragen van deze vragenlijst in de volgorde waarin ze gesteld zijn.

De vragenlijst bestaat uit zes ‘blokken’ met in totaal 52 vragen.

**INVULLEN:**

- U kruist bij elke vraag **één antwoord** aan, behalve wanneer duidelijk is aangegeven dat u meerdere antwoorden kunt aankruisen.
- Denk niet te lang na over een antwoord. Vul het antwoord in dat u het eerste in gedachten komt. Het gaat om uw mening; **elk antwoord is goed**.

**VERZENDEN NAAR :**

Sanquin, CLB,

OPZI, postvak 40,

antwoordnummer 814,

1000 SE Amsterdam (een postzegel is niet nodig).

**CONTACT:**

[opzi@amc.uva.nl](mailto:opzi@amc.uva.nl)

020-5123452 (Joke Koelewijn of Rina Siemons)

| Blok 1. Verloop zwangerschap |
| --- |

**NB.** De vragen in dit blok betreffen de zwangerschap, dus de periode tot het moment dat de weeën beginnen. De vragen over de bevalling volgen in blok 2.

**1. Hoe lang heeft de zwangerschap geduurd?** ……….weken en ……….dagen*

* Als u dit niet precies weet, kunt u invullen ………. dagen te vroeg / over tijd

**2. Door wie is de zwangerschap tot aan het begin van de weeën gecontroleerd?**

□ verloskundige □ gynaecoloog

□ verloskundige en gynaecoloog □ huisarts

**3. Bent u tijdens de zwangerschap – dus vóór het begin van de weeën –**

**opgenomen geweest in het ziekenhuis?**

□ nee

□ ja , …………. dagen, in verband met ……………………………….

..............................................

4. Hebben er verder medische ingrepen of extra controles plaatsgevonden

tijdens de zwangerschap, vóór het begin van de weeën?

□ nee

□ ja □ screeningsecho bij 18-20 weken

□ vruchtwaterpunctie

□ vlokkentest

□ extra bloedonderzoek

□ groeiecho’s

□ hartregistratie van het kind

□ anders, namelijk …………………………………..

…………………………...........................................

5. Bent u in deze zwangerschap geconfronteerd met een afwijkende testuitslag ?

□ nee

□ ja

Zo ja, om welke testuitslag ging het en hoe liep het verder af?

...........................................................................................................................................

...........................................................................................................................................

...........................................................................................................................................

6. Heeft één van de volgende situatie(s) zich voorgedaan tijdens de

zwangerschap?

Meerdere antwoorden zijn mogelijk.

□ ja, namelijk □ werkdruk/werkconflict

□ zelf ontslag/ontslag partner

□ verhuizing

□ relatieproblemen

□ sterfgeval

□ anders, ……………………………

……………………………………

□ geen

7 . Vindt u dat uw zwangerschap tot aan het begin van de weeën goed is

verlopen?

□ ja □ nee, niet zo goed: □ betreft mijzelf

□ betreft mijn kind

□ betreft mijn kind en mijzelf

Indien de zwangerschap minder goed is verlopen, kunt u dit dan toelichten? We hopen dat u toch bereid bent om ook de rest van de vragen zoveel mogelijk te beantwoorden.

...........................................................................................................................................

...........................................................................................................................................

**8. Hebben eventuele zorgen over uw zwangerschap geleid tot veranderingen in**

**uw leefwijze, zoals:**

□ geen veranderingen

□ wel veranderingen, namelijk:

U kunt meerdere zaken aankruisen.

□ wat meer roken □ wat ongezonder eten

□ wat meer alcoholgebruik □ iets slechter slapen

□ af en toe medicijngebruik □ anders……………………

| Blok 2. Verloop bevalling |
| --- |

**9. Op welke datum is/zijn uw kind(eren) geboren?** 

d d m m j j

10. Waar bent u uiteindelijk bevallen?

□ thuis □ ziekenhuis i.v.m. een medische reden

□ kraamkliniek/kraamhotel □ ziekenhuis op eigen verzoek

11. Welke verloskundige zorgverlener heeft uiteindelijk de bevalling begeleid?

□ verloskundige

□ gynaecoloog

□ huisarts

□ anders, namelijk …………………………………………..

12. Was er sprake van een medische ingreep rondom de bevalling?

□ nee Meerdere antwoorden zijn mogelijk.

□ ja, namelijk □ hartregistratie kind vóór geboorte

□ inleiding van de bevalling

□ vacuümextractie

□ tangverlossing

□ afgesproken keizersnede

□ spoedkeizersnede

□ bloedtransfusie moeder

□ placentaverwijdering onder

narcose

□ hechten onder narcose □ zuurstoftoediening kind

□ anders, nl:…………………………

13. Vindt u zelf dat uw bevalling goed is verlopen?

□ ja □ nee, niet zo goed: □ betreft mijzelf

□ betreft mijn kind(eren)

□ betreft mijn kind(eren) en

mijzelf

Indien de bevalling minder goed is verlopen, kunt u dit dan toelichten?

...........................................................................................................................................

...........................................................................................................................................

| Blok 3. Verloop na de bevalling |
| --- |

14. Was er direct na de bevalling medische behandeling nodig bij uw kind(eren)?

□ nee

Meerdere antwoorden zijn mogelijk.

□ ja, namelijk □ opname intensive care (NICU):

…………….. dagen

□ opname kinderafdeling:

……………..dagen □ bloedtransfusie(s) kind

□ fototherapie (onder de lamp)

□ andere medische behandeling,

nl:…………………………………..

15. Was er medische behandeling nodig bij uzelf?

□ nee

Meerdere antwoorden zijn mogelijk.

□ ja, namelijk □ alsnog opname:

…………….. dagen

□ curettage

□ bloedtransfusie voor uzelf

□ toediening antibiotica

□ andere medische behandeling,

nl:…………………………………

16. Vindt u zelf dat de periode na de bevalling goed is verlopen voor u en uw
 kind(eren)?

□ ja □ nee, niet zo goed: □ betreft mijzelf

□ betreft mijn kind(eren)

□ betreft mijn kind(eren) en

mijzelf

Indien de periode na de bevalling minder goed is verlopen, kunt u dit dan toelichten?

...........................................................................................................................................

...........................................................................................................................................

17. Vindt u zelf dat het op dit moment goed gaat met u en uw kind(eren)?

LET OP: *wanneer u deze vragenlijst later dan 2 weken na de bevalling invult, denk dan bij het invullen terug aan hoe het 2 weken na de bevalling ging.*

□ ja □ nee, niet zo goed: □ betreft mijzelf

□ betreft mijn kind(eren)

□ betreft mijn kind(eren) en

mijzelf

Indien het minder goed gaat met u en/of uw kind(eren), kunt u dit dan toelichten?

...........................................................................................................................................

...........................................................................................................................................

#

| Blok 4. Beleving van uw zwangerschap |
| --- |

Zwangerschap en bevalling zijn soms een spannende periode. Soms maken vrouwen zich zorgen over zichzelf of over hun kind. In dit blok vragen we in hoeverre u zich zorgen hebt gemaakt tijdens de zwangerschap, de bevalling en daarna.

Bij de volgende vragen is het de bedoeling dat u uw antwoord aangeeft op een schaal. U kunt dat doen door een kruisje te zetten op de lijn op het punt dat het beste met uw mening overeenkomt.

**VOORBEELDVRAAG:**

**De informatie over de bereikbaarheid van mijn verloskundige zorgverlener in het weekend vind ik:**

heel zeer

onduidelijk onduidelijk duidelijk duidelijk

*Dit kruisje in de voorbeeldvraag geeft aan dat u de informatie redelijk duidelijk vindt.*

***Hierna volgen de echte vragen.***

Ga voor de eerste twee vragen in gedachten terug naar de tweede helft van de zwangerschap (vanaf de 18^e^ week).

**18. Geef aan in hoeverre u zich tijdens de zwangerschap zorgen hebt gemaakt**

**over uzelf.**

helemaal niet een beetje nogal heel erg

**19. Geef aan in hoeverre u zich tijdens de zwangerschap zorgen hebt gemaakt**

**over uw kind(eren).**

helemaal niet een beetje nogal heel erg

**20. Geef aan in hoeverre u zich tijdens de bevalling zorgen hebt gemaakt over**

**uzelf.**

helemaal niet een beetje nogal heel erg

**21. Geef aan in hoeverre u zich tijdens de bevalling zorgen hebt gemaakt over uw**

**kind(eren).**

helemaal niet een beetje nogal heel erg

**22. Geef aan in hoeverre u zich sinds de geboorte van uw kind(eren) zorgen hebt**

**gemaakt over uzelf.**

helemaal niet een beetje nogal heel erg

**23. Geef aan in hoeverre u zich sinds de geboorte van uw kind(eren) zorgen hebt**

**gemaakt over uw kind(eren).**

helemaal niet een beetje nogal heel erg

**24. Geef aan in hoeverre u zich op dit moment zorgen maakt over uzelf.**

LET OP: *wanneer u deze vragenlijst later dan 2 weken na de bevalling invult, denk dan bij het invullen terug aan hoe het 2 weken na de bevalling ging.*

helemaal niet een beetje nogal heel erg

**25. Geef aan in hoeverre u zich op dit moment zorgen maakt over uw kind(eren).**

LET OP: *wanneer u deze vragenlijst later dan 2 weken na de bevalling invult, denk dan bij het invullen terug aan hoe het 2 weken na de bevalling ging.*

helemaal niet een beetje nogal heel erg

#

| Blok 5. Bloedgroep-antistoffen |
| --- |

In de vorige vragenlijst zijn over dit onderwerp vele vragen gesteld. Er werden vragen gesteld over uw kennis over bloedgroep-antistoffen en naar uw beleving ten aanzien van het routine bloedonderzoek naar de antistoffen. Ook vroegen we uw mening over de informatievoorziening door zorgverleners.

**26. Had u in uw geval nog meer informatie willen krijgen over bloedgroep-**

**antistoffen en de onderzoeken en behandelingen die daarbij horen?**

□ nee

□ ja: *Op welke manier? Over welke onderwerpen?*

U kunt meerdere zaken aankruisen: U kunt meerdere zaken aankruisen:

□ schriftelijke informatie □ bloedonderzoeken

□ mondelinge informatie □ echoscopieën

□ via een speciaal telefoonnummer □ vruchtwaterpuncties

□ via een Internetsite □ transfusie in de baarmoeder

□ gevolgen antistoffen voor het kind

□ behandeling kind na de geboorte

□ gevolgen antistoffen voor mijzelf

□ een volgende zwangerschap

□ anders ……………………………… □ anders ………………………………

*Ter afsluiting van dit onderdeel stellen we nog enkele vragen waarin u uw totale oordeel kunt geven over het bloedonderzoek op bloedgroep-antistoffen.*

**27. In hoeverre is het bloedonderzoek op bloedgroep-antistoffen voor uzelf**

**psychisch belastend geweest?**

helemaal niet een beetje nogal heel erg

**28. Vindt u dat het bloedonderzoek op bloedgroep-antistoffen nuttig is geweest**

**voor u en voor uw kind?**

helemaal niet een beetje nogal heel erg

**29. Als u voor uzelf de psychische belasting van het bloedonderzoek op**

**bloedgroep-antistoffen afweegt tegen het nut hiervan, wat is dan uw totale**

**oordeel over dit bloedonderzoek?**

totaal weinig redelijk heel erg

onbelangrijk belangrijk belangrijk belangrijk

**30. Als u voor iemand anders (bijvoorbeeld een goede vriendin) de psychische**

**belasting van het bloedonderzoek op bloedgroep-antistoffen afweegt tegen**

**het nut hiervan, wat is dan uw totale oordeel over dit bloedonderzoek?**

totaal weinig redelijk heel erg

onbelangrijk belangrijk belangrijk belangrijk

**31. Zijn er nog zaken over het bloedonderzoek op bloedgroep-antistoffen die niet**

**aan de orde zijn geweest? Hebt u tips om/hoe het standaard bloedonderzoek**

**(dus niet deze studie) te verbeteren?**

…………………………………………………………………………………………………….

…………………………………………………………………………………………………….

…………………………………………………………………………………………………….

| Blok 6. Zelf beschrijving |
| --- |

*Hieronder vindt u een aantal uitspraken, die mensen hebben gebruikt om zichzelf te beschrijven. Lees iedere uitspraak door en zet dan een kringetje om het cijfer rechts van die uitspraak om aan te geven hoe u zich nu voelt, dus nu op dit moment. Er zijn geen goede of slechte antwoorden. Denk niet te lang na en geef uw eerste indruk, die is meestal de beste. Het gaat er dus om dat u weergeeft wat u op dit moment voelt.*

geheel een tamelijk zeer

niet beetje veel veel

32. Ik voel me kalm . . . . . . . . . . . . . . . . . 1 2 3 4

33. Ik voel me veilig . . . . . . . . . . . . . . . . . 1 2 3 4

34. Ik ben gespannen . . . . . . . . . . . . . . . . . 1 2 3 4

35. Ik voel me onrustig . . . . . . . . . . . . . . . . 1 2 3 4

36. Ik voel me op mijn gemak . . . .. . . . . . . . . 1 2 3 4

37. Ik ben in de war. . . . . . . . . . . . . . . . . . 1 2 3 4

38. Ik pieker over nare dingen die kunnen gebeuren 1 2 3 4

39. Ik voel me voldaan . . . . . . . . . . . . . . . . 1 2 3 4

40. Ik ben bang . . . . . . . . . . . . . . . . . . . 1 2 3 4

41. Ik voel me aangenaam. . . . . . . . . . . . . . 1 2 3 4

42. Ik voel me zeker . . . . . . . . . . . . . . . . . 1 2 3 4

43. Ik voel me nerveus . . . . . . . . . . . . . . . . 1 2 3 4

44. Ik ben zenuwachtig . . . . . . . . . . . . . . . 1 2 3 4

45. Ik ben besluiteloos . . . . . . . . . . . . . . . . 1 2 3 4

46. Ik ben ontspannen . . . . . . . . . . . . . . . . 1 2 3 4

47. Ik voel me tevreden . . . . . . . . . . . . . . . 1 2 3 4

48. Ik maak me zorgen . . . . . . . . . . . . . . . . 1 2 3 4

49. Ik voel me gejaagd . . . . . . . . . . . . . . . . 1 2 3 4

50. Ik voel me evenwichtig . . . . . . . . . . . . . . 1 2 3 4

51. Ik voel me prettig . . . . . . . . . . . . . . . . . 1 2 3 4

geheel een tamelijk zeer

niet beetje veel veel

U bent nu klaar met het invullen van deze vragenlijst.

**52. Heeft het invullen van de vragenlijst(en) over bloedgroep-antistoffen nog**

**invloed gehad op de zorgen die u zich eventueel maakte over bloedgroep-**

**antistoffen?**

veel minder wat minder wat meer veel meer

zorgen zorgen zorgen zorgen

Op welke datum hebt u deze lijst ingevuld? 200 

d d m m j j j j

Stuur deze vragenlijst direct naar ons terug in de bijgevoegde antwoordenvelop.

Hartelijk dank voor de medewerking.

Als u geen antwoordenvelop heeft, kunt u de vragenlijst zonder postzegel terugsturen naar:

Sanquin, CLB

OPZI-onderzoek, postvak 40

Antwoordnummer 814

1000 SE AMSTERDAM

Ook kunt u ons bellen, zodat wij u alsnog een antwoordenvelop kunnen toesturen:

020- 5123452 (Joke Koelewijn of Rina Siemons)
